# Cytomegalovirus retinitis in a seronegative patient with systemic lupus erythematosus on immunosuppressive therapy

**DOI:** 10.1007/s12348-010-0017-3

**Published:** 2011-04-03

**Authors:** Aditya Kelkar, Jai Kelkar, Shreekant Kelkar, Shilpa Bhirud, Jyotirmoy Biswas

**Affiliations:** 1 National Institute of Ophthalmology, 1187/30 off Ghole Road, Shivajinagar, Pune, 411005 Maharashtra India; 2 Sankara Nethralaya, 18 College Road, Chennai, 600006 Tamil Nadu India

**Keywords:** CMV retinitis, Seronegative, SLE, Grade 4 lupus nephritis

## Abstract

**Aim:**

The purpose of this study is to report a rare case of cytomegalovirus (CMV) retinitis in a seronegative patient with systemic lupus erythematosus (SLE) on immunosuppressive therapy.

**Methods:**

A seronegative patient with SLE who was on immunosuppressive therapy developed CMV retinitis. The immunosuppressive therapy was tapered, and the patient was given intravitreal ganciclovir and foscarnet in addition to systemic ganciclovir. The follow-up visits were documented.

**Result:**

The patient responded to the treatment and there was complete resolution.

**Conclusion:**

CMV retinitis is a rapidly progressive condition and patients on immunosuppressive therapy should be referred to an ophthalmologist for periodic check-up for early diagnosis and treatment of this devastating ophthalmic condition. For clinically resistant CMV retinitis in seronegative patients with SLE, a combination therapy of intravitreal foscarnet with oral and intravenous ganciclovir is useful.

## Introduction

Systemic lupus erythematosus (SLE) is not generally considered a predisposing factor for cytomegalovirus (CMV) retinitis. Immunosuppressive therapy may have a role in the development of CMV retinitis in these patients [1]. We report a case of bilateral CMV retinitis in a seronegative patient with systemic lupus erythematosus on immunosuppressive therapy for grade 4 lupus nephritis.

## Case report

In 2009, a 31-year-old female, known case of systemic lupus erythematosus with stage IV lupus on immunosuppressive therapy, presented with floaters and gradual painless progressive diminution of vision more in the right eye than left eye since 1 week. Two years earlier, in 2007, the patient had developed fever, skin rashes, nausea, breathing difficulty, polyarthralgias and myalgias. She had been admitted to a hospital and had been diagnosed as SLE based on clinical presentation and investigation results which were as follows: anti-DNA positive (3.68); proteinuria, 4+; raised 24-h urine protein, 5,432 mg/24 h (21.3–119.6 mg/24 h); ANA positive (1.56); Ro-52 positive; raised C-reactive protein level, 30 mg/L. The renal biopsy showed diffuse proliferative glomerulonephritis with capillary wall thickening consistent with lupus nephritis (WHO class IV) with activity index, 04; chronicity index, 01. She had been given intravenous methyl prednisolone 1 g daily for 1 week, azathioprine tablet 50 mg twice a day, hydroxychloroquine tablet 200 mg twice a day and furosemide tablet 10 mg three times a day. Systemic steroids 1 mg/kg tapering every 7 days had been started after the course of intravenous methyl prednisolone. The patient improved and had been discharged.

She was regularly followed up and steroid maintenance dose of 10 mg alternated with 5 mg continued in addition to azathioprine 100 mg twice a day, mycophenolate mofetil 500 mg twice a day, hydroxychloroquine 200 mg once a day, furosemide 10 mg twice a day and calcium supplement 500 mg twice a day. Renal function tests, hemogramme, ESR, 24-h urinary proteins, liver function tests were done periodically. She had no ophthalmic complaints till 2009. In 2009, the patient developed high-grade fever, urinary tract infection and oral candidiasis. She was admitted in the hospital and treated for the same. She was discharged after 1 week. At the time of discharge, the patient started complaining of diminution of vision and floaters in the right eye more than the left eye. She was then referred to the ophthalmologist.

**Fig. 1:**
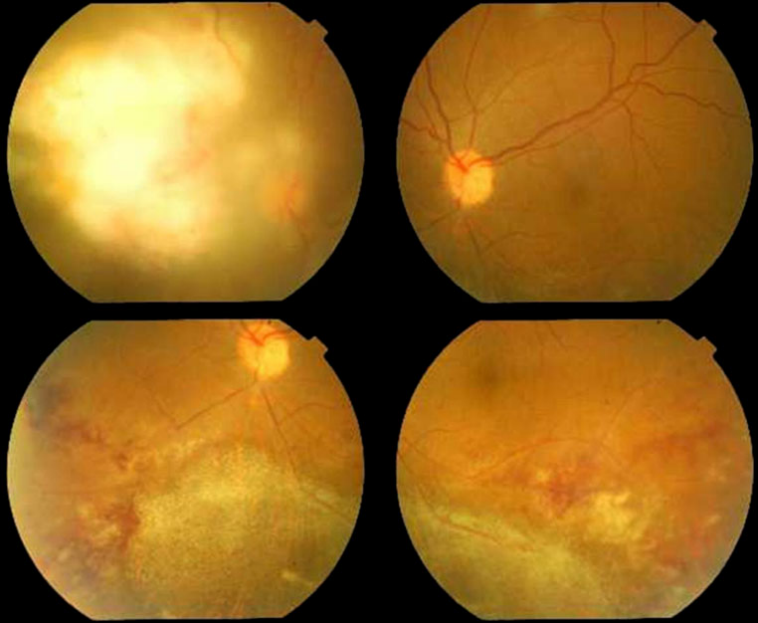
The right eye showed a ten-disc-diameter size yellowish white lesion with hazy irregular border at the posterior pole. There was mild vitreous haze. The left eye showed presence of perivascular exudates, retinal haemorrhages along the inferior vessels with mild vitreous haze

On ocular examination, the vision in the right eye was finger counting 1 per metre and in the left eye it was 20/60.The anterior segment and intraocular pressures were normal in both eyes .On fundus examination, the right eye showed a ten-disc-diameter size yellowish white lesion with hazy irregular border at the posterior pole. There was mild vitreous haze. The left eye showed presence of perivascular exudates, retinal haemorrhages along the inferior arcade with mild vitreous haze (Fig. 1). A clinical diagnosis of bilateral CMV retinitis was made. There was no evidence of systemic CMV infection.

**Fig. 2:**
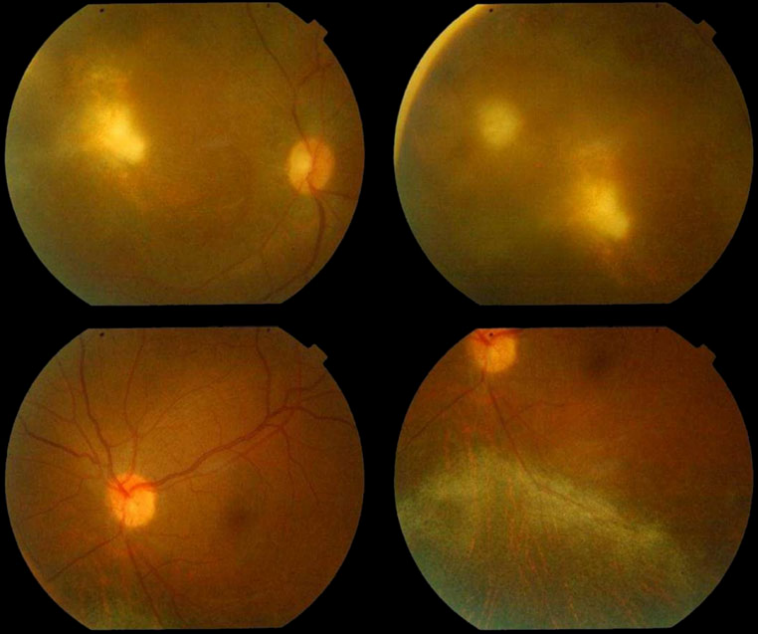
Haemorrhages and exudates along the inferior arcade disappeared in the left eye. The right eye lesion, although, became smaller

**Fig. 3:**
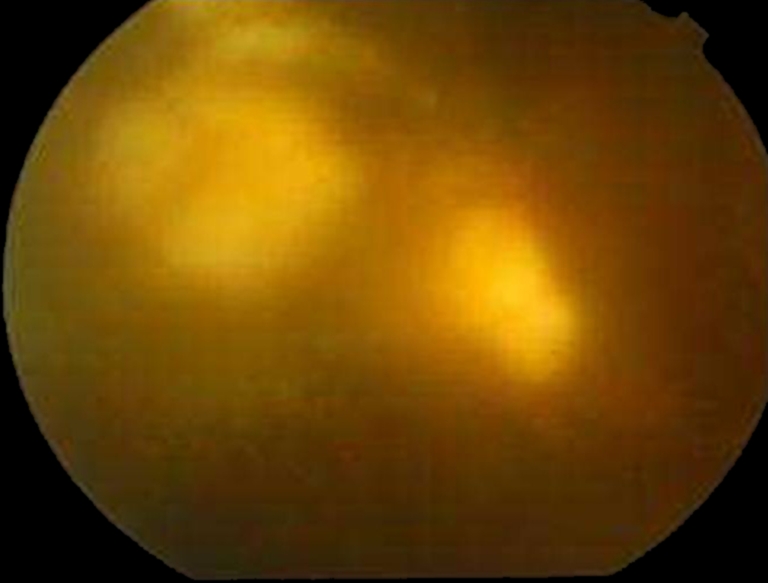
The lesions in the right eye started increasing and the borders of the lesions got fuzzier

**Fig. 4:**
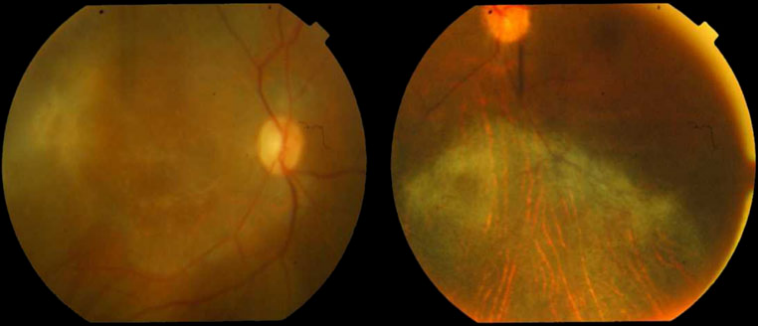
The lesions in both eyes healed completely

Results of an enzyme-linked immunosorbent assay test for antibodies to human immunodeficiency virus, hepatitis B and C were negative, CMV IgM antibody was negative and IgG was positive at an immune status ratio of 2.87 (0–0.90), toxoplasma IgG, IgM and VDRL was negative. Blood culture was negative for CMV. The absolute lymphocyte count was 680/mm^3^, total leucocytic count, 10,700/mm^3^; CD4, 81. The polymerase chain reaction of the aqueous sample was positive for CMV retinitis and negative for herpes simplex virus and herpes zoster virus and toxoplasmosis. After consultation with the rheumatologist, mycophenolate mofetil was discontinued; azathioprine dose was reduced to 50 mg twice daily and oral steroids were tapered to 5 mg daily. The patient was started on intravenous ganciclovir 250 mg twice daily for 3 weeks and oral valganciclovir 900 mg twice daily for 8 weeks. Intravitreal ganciclovir 0.05 mg/0.1 ml weekly eight injections in each eye were given. At 2 months, vision improved to counting finger 2 m in the right eye and six ninths in the left eye. Haemorrhages and exudates along the inferior arcade disappeared in the left eye. The right eye lesion, although, became smaller significantly but did not disappear completely (Fig. 2); hence, oral valganciclovir and intravitreal weekly ganciclovir was continued. Within 15 days, the lesions in the right eye started increasing and the borders of the lesions got fuzzier (Fig. 3). The vision was counting finger 1 metre. For this clinically resistant CMV retinitis, intravitreal 2.4 mg/0.1 ml foscarnet was given in the right eye biweekly and weekly in the left eye for 3 weeks. In addition, the intravenous ganciclovir 250 mg was given twice daily and the oral valganciclovir 900 mg twice daily was continued as before. At the end of 3 weeks, the lesions healed completely in the right eye (Fig. 4).The vision in the right eye was 2 m and in the left eye was 20/20.Oral valganciclovir was discontinued 1 month after complete resolution of the ocular lesions. No recurrences were noted up to 6 months.

## Discussion

CMV retinitis in patients without HIV infection have a clinical course similar to that in patients with AIDS treated with highly active antiretroviral therapy. A substantial number of non-HIV patients do not require long-term anti-CMV therapy after adjustment of immunomodulatory therapy [2].

A patient with inactive SLE on mild immunosuppression without systemic manifestations of CMV infection has been reported before [3]. SLE, per se, does not seem to be the primary predisposing factor for the development of CMV retinitis [1]. Cytomegalovirus is a ubiquitous virus, and primary exposure usually occurs at an early age. However, clinical signs of infection usually do not manifest in immunocompetent individuals. Adult-onset CMV disease may occur as a result of primary infection, reinfection or activation of a latent infection [4].

At the time when our patient was diagnosed as having CMV retinitis, she was not experiencing an acute lupus flare, and therefore it was possible to discontinue mycophenolate mofetil and reduce the dose of azathioprine and oral steroids after consultation with the physician.

Intravitreal injections of ganciclovir are useful for the non-HIV immunocompromised patients with CMV retinitis [5]. Therapy with a combination of intravitreal foscarnet and intravenous ganciclovir in clinically resistant CMV retinitis in patients with acquired immunodeficiency syndrome has been reported [6].

In our patient with clinically resistant CMV retinitis, discontinuation of mycophenolate mofetil and reduction of the dose of Azathioprine and oral steroids in addition to systemic and intravitreal ganciclovir therapy brought the disease process well under control, but the lesions in the right eye showed signs of resurgence and therefore a combination of intravitreal foscarnet along with intravenous ganciclovir and oral valganciclovir were given. The lesions resolved completely.There was no evidence of reactivation up to 6 months.

## Conclusion

CMV retinitis is a rapidly progressive condition and patients on immunosuppressive therapy should be referred to an ophthalmologist for periodic check-up for early diagnosis and treatment of this devastating ophthalmic condition. For clinically resistant CMV retinitis in seronegative patients with SLE, a combination therapy of intravitreal foscarnet with oral and intravenous ganciclovir is useful.
